# Simvastatin suppresses breast cancer cell proliferation induced by senescent cells

**DOI:** 10.1038/srep17895

**Published:** 2015-12-14

**Authors:** Su Liu, Harpreet Uppal, Marco Demaria, Pierre-Yves Desprez, Judith Campisi, Pankaj Kapahi

**Affiliations:** 1 Buck Institute for Research on Aging, Novato, CA 94945, USA; 2 Santa Rosa Junior College, Santa Rosa, CA 95401, USA; 3 California Pacific Medical Center, Research Institute, San Francisco, CA 94107, USA; 4 Life Sciences Division, Lawrence Berkeley National Laboratory, Berkeley, CA 94720, USA

## Abstract

Cellular senescence suppresses cancer by preventing the proliferation of damaged cells, but senescent cells can also promote cancer though the pro-inflammatory senescence-associated secretory phenotype (SASP). Simvastatin, an HMG-coA reductase inhibitor, is known to attenuate inflammation and prevent certain cancers. Here, we show that simvastatin decreases the SASP of senescent human fibroblasts by inhibiting protein prenylation, without affecting the senescent growth arrest. The Rho family GTPases Rac1 and Cdc42 were activated in senescent cells, and simvastatin reduced both activities. Further, geranylgeranyl transferase, Rac1 or Cdc42 depletion reduced IL-6 secretion by senescent cells. We also show that simvastatin mitigates the effects of senescent conditioned media on breast cancer cell proliferation and endocrine resistance. Our findings identify a novel activity of simvastatin and mechanism of SASP regulation. They also suggest that senescent cells, which accumulate after radio/chemo therapy, promote endocrine resistance in breast cancer and that simvastatin might suppress this resistance.

Cellular senescence is a complex stress response that is activated by a variety of stresses, including dysfunctional telomeres, DNA damage and oncogene activation^1^. Salient features of senescent cells include cell enlargement, activity of the senescence-associated β-galactosidase (SA-β-gal)^2^, and persistent DNA damage foci^3^. In addition, senescent cells acquire a complex senescence-associated secretory phenotype (SASP) – the secretion of numerous cytokines, chemokines, growth factors and proteases^4^,^5^,^6^. Senescent cells also secrete the alarmin HMGB1, which can initiate an inflammatory response^7^. It is now clear that cellular senescence can be beneficial or deleterious, depending on the age and physiological state of the organism.

On the positive side, the senescence response can be a formidable barrier to cancer progression by halting the growth of damaged, potentially oncogenic cells^8^. In addition, senescent cells are induced at sites of tissue damage and during certain stages of embryogenesis where they, and particularly certain SASP factors they secreted, appear to be important for optimal wound healing and development^9^,^10^. On the negative side, senescent cells increase with age and at sites of age-related pathology, where the loss of proliferative capacity and SASP are thought to drive a number of aging phenotypes^1^. Notably, senescent fibroblasts can promote epithelial cell growth and tumorigenesis in a cell non-autonomous manner^11^, owing in part to certain pro-inflammatory SASP factors such as IL-6, IL-8 and CXCL-1^12^.

The ability of the SASP to promote inflammation and cancer progression suggests it should be possible to identify drugs that can suppress its activities. Indeed, in a screen of FDA approved drugs we identified glucocorticoids as potent suppressors of selected components of the SASP^13^. Subsequently, a family of drugs, statins, caught our attention owing to their reported anti-inflammatory activities^14^. Statins are competitive inhibitors of 3-hydroxy-3-methylglutaryl coenzyme A reductase (HMGCR), the rate limiting enzyme in cholesterol synthesis, which catalyzes the conversion of HMG-CoA to mevalonate^15^. Statins are widely used as cholesterol-lowering drugs, and significantly reduce the risk of coronary heart disease and other vascular events in a large number of patients^16^. Moreover, increasing evidence indicates that the clinical benefits of statins extend beyond lowering blood cholesterol levels.

Simvastatin is a statin that can reduce the expression of pro-inflammatory cytokines such as IL-6, IL-8, and MCP-1 both in culture and *in vivo*^17^,^18^. Selected other statins have similar anti-inflammatory effects^19^. Interestingly, certain statins modestly ameliorate cell cycle arrest and SA-β-gal expression by mesenchymal stem cells^20^ and chondrocytes^21^, suggesting they might suppress certain senescent phenotypes. On the other hand, some statins induce SA-β-gal expression and modestly retard growth by myofibroblasts^22^ and prostate cancer cells^23^. Thus, the effects of statins appear to vary with both drug and cell type with respect to cell proliferation and one marker (SA-β-gal) of senescence. Virtually nothing is known about whether statins affect the SASP.

In addition to lowering cholesterol synthesis, statins also dampen the formation of intermediate metabolites of the mevalonate pathway, including the isoprenoids geranylgeranyl pyrophosphate (GGPP) and farnesyl pyrophosphate (FPP), which are the donors for protein prenylation. Known prenylated proteins include the major nuclear lamina protein lamin A, members of the Ras superfamily of signal transduction switches, and several protein kinases^24^.

The Rho GTPases (a subgroup of the Ras superfamily) function as molecular switches for diverse cellular functions such as cell motility, adhesion and proliferation^25^. Among the ~20 Rho family GTPases, Rho A, Rac1 and Cdc42 are the most well studied and have been linked to cellular senescence, although the molecular mechanism explaining this link is unclear^26^,^27^. Like most other Rho family members, Rho A, Rac1 and Cdc42 cycle between a GTP-bound active form and GDP-bound inactive form, and protein prenylation is critical for activation.

Several meta-analyses suggest that the long-term statin use reduces the risk of several types of cancers, including hepatocellular carcinoma^28^, esophageal cancer^29^ and prostate cancer^30^. In the case of breast cancer, epidemiological and clinical studies have not identified a strong relationship between statin use and cancer incidence^31^, but the use of simvastatin was associated with a reduced risk of recurrence among Danish women diagnosed with stage I-III breast carcinoma^32^. The effects of statins might depend on cancer subtypes and/or stage, patient gender and/or age, treatment time and type of statin (lipophilic or hydrophilic).

Breast cancer is the most common cancer among women in western nations. Approximately 70% of breast tumors are estrogen receptor (ER) positive. Treatment of these tumors with estrogen antagonists significantly reduces disease progression, but resistance to endocrine treatment eventually occurs in a substantial proportion of patients^33^. Some evidence suggests that overactive growth factor or inflammatory signaling might contribute to this resistance to endocrine therapy^34^.

Here, we show that simvastatin reduces the SASP, without affecting other senescence-associated phenotypes, by suppressing the activities of Rac1 and Cdc42. This suppression appeared to be achieved through the reduction of isoprenoid intermediates of the mevalonate pathway and subsequently impaired protein prenylation. We show that simvastatin mitigates the ability of senescent cells to stimulate the proliferation of breast cancer cells in culture, and, further, that the SASP promotes endocrine resistance in breast cancer cells, which is also mitigated by simvastatin. Our results identify a novel activity of simvastatin, and a new mechanism for the pleiotropic anti-cancer effects of simvastatin.

## Results

### Simvastatin reduces the SASP

**Figure 1:**
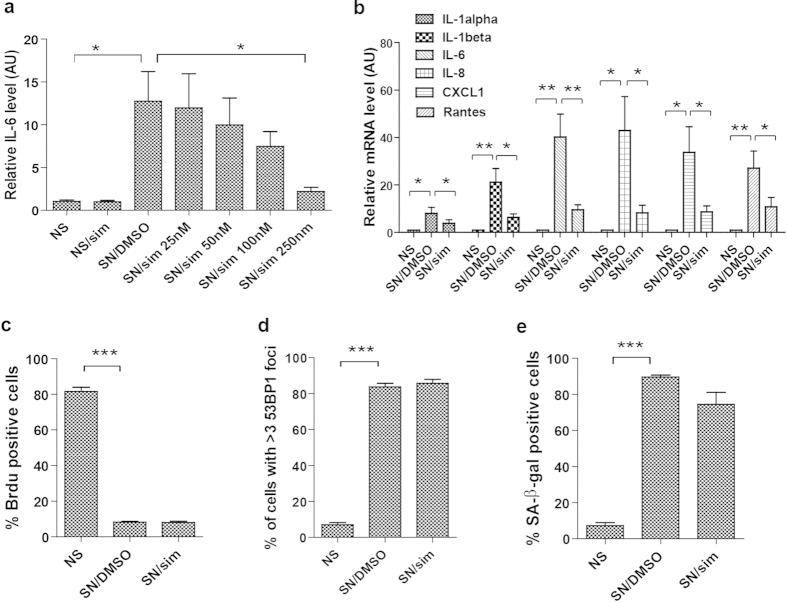
Simvastatin strongly reduces the SASP of human fibroblasts. (**a**) Non-senescent (NS) or senescent (SN) HCA2 fibroblasts were incubated in media containing the indicated concentrations of simvastatin (sim) or vehicle (DMSO). Cells were given simvastatin or DMSO immediately after irradiation and conditioned media (CM) were collected and analyzed 9 d later by ELISA for IL-6. Cells remained on the plate at the time of collection were counted for normalization. Shown are IL-6 levels relative to that of NS. (**b**) NS or SN cells were given 250 nM simvastatin or DMSO. 9 d later, mRNA was extracted for quantitative PCR analysis of the indicated genes, which were normalized to actin mRNA. (**c**) NS or SN cells described in (**b**) were given BrdU for 24 hrs, fixed and immunostained for nuclear BrdU; the percentage of BrdU-positive cells as scored manually. (**d**) NS or SN cells described in (**b**) were immunostained for 53BP1. The percentage of cells with >3 53BP1 nuclear foci was manually counted. (**e**) NS or SN cells described in (**b**) were stained for SA-β gal. The percentage of SA-β gal-positive cells was manually counted. (*p < 0.05, **p < 0.01, ***p < 0.001).

To study the effect of simvastatin, we induced senescence in normal human fibroblasts HCA2 by ionizing radiation (IR; 10 Gy), added varying concentrations of simvastatin immediately after IR, and maintained the cells in medium containing the drug for 9 d (Supplementary Figure 1a). On the 9th day, we incubated the cells in serum-free medium without simvastatin, collected the conditioned medium (CM) 24 hrs later, and determined the levels of IL-6, a prominent SASP component^5^, by ELISA. Simvastatin reduced IL-6 secretion by senescent cells in a dose dependent manner, with significant suppression (~75%) at 250 nM (Fig. 1a). This dose is within the range of serum concentrations used for standard cholesterol lowering in patients^35^. Higher doses of simvastatin resulted in cell death (Supplementary Figure 1b). Much of the SASP is due to increased mRNA abundance^5^. To determine whether simvastatin altered transcript levels or whether its effects were limited to IL-6, we determined the mRNA levels of several SASP factors after treating senescent cells with simvastatin. Simvastatin decreased IL-1α, IL-1β, IL-6, IL-8, CXCL-1, and Rantes mRNA expression (Fig. 1b), suggesting it is a broad suppressor of the SASP.

IR arrests cell proliferation within 24 hrs, while it takes 3–4 days for the SASP to develop and 7–10 days for full establishment^3^,^5^. Efficient suppression of the SASP required that simvastatin be added to cells immediately after IR, and that it be present at least 24 hrs before CM collection. Pretreatment before IR, or early withdrawal (i.e., treatment terminated 48 hrs before collection), failed to suppress the SASP (Supplementary Figure 1c).

Simvastatin partly prevented the typical senescence-associated enlarged morphology (Supplementary Figure 1d), but had no significant effects on the growth arrest (Fig. 1c; Supplementary Figure 1e), presence of persistent DNA damage foci (Fig. 1d; Supplementary Figure 1f), or fraction of cells that expressed the SA-β-gal (Fig. 1e; Supplementary Figure 1d). We confirmed our findings using another human fibroblast cell strain WI-38. We found that simvastatin also reduced IL-6 protein secretion (Supplementary Figure 2a) and IL-6, CXCL-1, and SFRP1 mRNA level (Supplementary Figure 2b), without affecting growth arrest (Supplementary Figure 2c) and SA-β-gal activity (Supplementary Figure 2d). Moreover, simvastatin reduced IL-6 and CXCL-1 mRNA expression in oncogene H-Ras induced senescent cells, without affecting SA-β-gal activity or the expression of p16 and p21 (Supplementary Figure 3a and b).

Thus, simvastatin suppressed the expression of several SASP components and the senescence-associated morphology, without affecting other senescent phenotypes, most notably the tumor suppressive growth arrest.

### Simvastatin suppresses the SASP by inhibiting protein prenylation

**Figure 2:**
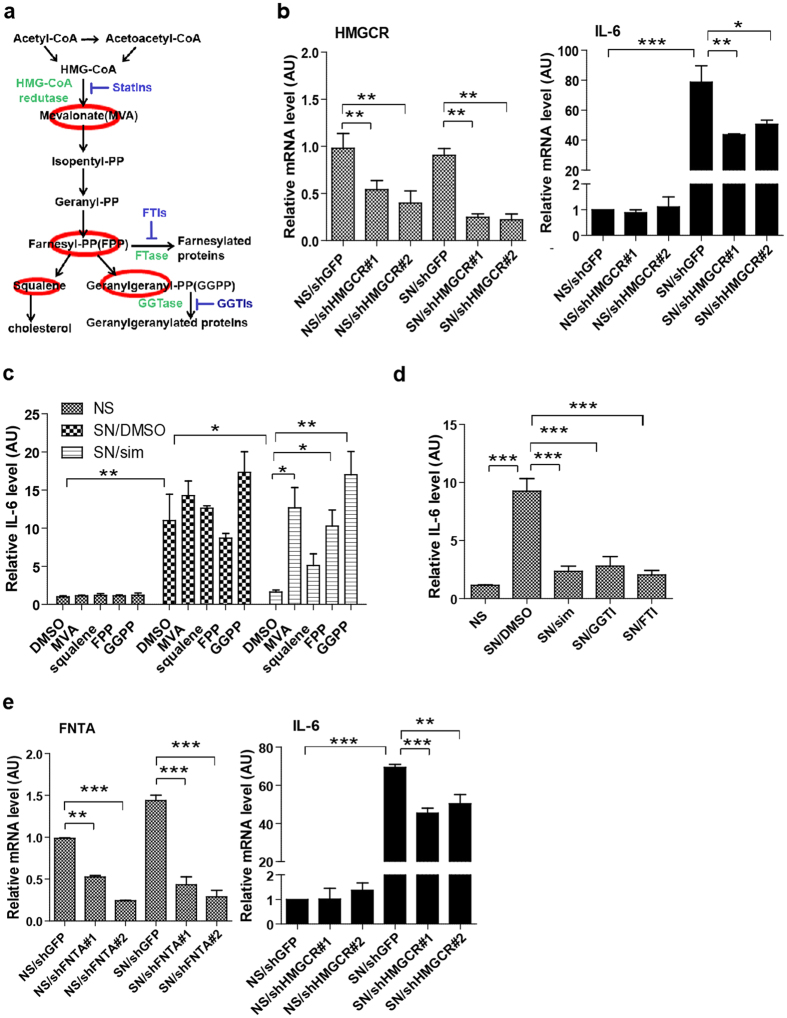
Simvastatin suppresses the SASP by inhibiting protein prenylation. (**a**) Simplified schematic of the mevalonate pathway. The intermediate metabolites that were added to simvastatin-treated cells in (**b**) are circled in red. (**b**) NS or SN fibroblasts were infected with lentiviruses expressing an shRNA against HMG CoA reductase (shHMGCR) or a control shRNA (shGFP), and selected. 9 d later, mRNA was collected for qPCR analysis for HMGCR (left panel) or IL-6 (right panel). Actin was used as a control for RNA quantity. (**c**) NS or SN fibroblasts were treated with DMSO or 250 nM simvastatin in the presence or absence of the mevalonate pathway metabolites for 7 d. CM were collected and analyzed by ELISA for IL-6 as described above. (**d**) NS or SN fibroblasts were treated with DMSO, 250 nM simvastatin, 20 μM geranylgeranyl transferase inhibitor (GGTI), or 5 μM farnesyltransferase inhibitor (FTI) for 9 d. CM were collected and analyzed by ELISA for IL-6 as described above. (**e**) NS or SN fibroblasts were infected with a lentivirus expressing an shRNA against the α subunit of farnesyl transferase/geranylgeranyltransferase (shFNTA) or a control shRNA (shGFP), and selected. 9 d later, mRNA was collected for qPCR analysis of FNTA (left panel) or IL-6 (right panel) mRNAs. Actin was used as a control for RNA quantity. (*p < 0.05, **p < 0.01,***p < 0.001).

Simvastatin reduces cholesterol production by inhibiting the first and the rate-limiting enzyme of the mevalonate pathway: HMG-CoA reductase (HMGCR). Other than lowering cholesterol synthesis, simvastatin also reduces the formation of isoprenoid metabolites, including geranylgeranyl pyrophosphate (GGPP) and farnesyl pyrophosphate (FPP) (Fig. 2a), which are donors for protein prenylation. To determine whether simvastatin reduces IL-6 secretion by senescent cells by inhibiting HMGCR, we used lentiviruses to express short hairpin (sh) RNAs designed to deplete cells of HMGCR. Quantitative PCR confirmed that two distinct shRNAs reduced HMGCR mRNA (Fig. 2b, left panel), and also significantly reduced IL-6 mRNA levels in senescent cells (Fig. 2b, right panel).

To determine whether simvastatin suppressed the SASP through lowering cholesterol or reducing the isoprenoid intermediates, we tested whether IL-6 secretion could be reverted by administering metabolites of the mevalonate pathway: mevalonate (MVA) (200 μM), squalene (10 μM), FPP (10 μM) or GGPP (10 μM)^36^. MVA, FPP and GGPP each restored IL-6 secretion to simvastatin-treated senescent cells to levels similar to those of vehicle-treated senescent cells, whereas squalene was largely ineffective at restoring IL-6 secretion (Fig. 2c), suggesting that cholesterol lowering is not required for IL-6 suppression by simvastatin in senescent cells. MVA, FPP or GGPP did not appreciably alter IL-6 secretion by non-senescent (mock-irradiated) cells or senescent cells treated with vehicle. Thus, inhibition of isoprenoid intermediates is critical for the ability of simvastatin to reduce IL-6 secretion by senescent cells.

Isoprenoid intermediates of the mevalonate pathway are essential for protein prenylation^37^. To determine whether protein prenylation was required for the SASP, we treated senescent cells with the geranylgeranyltransferase inhibitor GGTI-2133 or the farnesyltransferase inhibitor FTI-277, and examined IL-6 secretion by senescent cells. GGTI-2133 (20 μM) or FTI-277 (5 μM) reduced IL-6 secretion 75% (Fig. 2d), suggesting that protein prenylation is essential for this SASP feature. Geranylgeranyltransferase and farnesyltransferase share an α subunit but have distinct β subunits^38^. To confirm that protein prenylation was essential for IL-6 secretion by senescent cells, we assessed IL-6 mRNA levels after depleting FNTA, the α subunit that is shared by geranylgeranyltransferase and farnesyltransferase. shRNAs targeting FNTA (shFNTA), but not a control shRNA (shGFP), downregulated FNTA expression (Fig. 2e, left panel) and significantly suppressed IL-6 mRNA levels in senescent cells (Fig. 2e, right panel), suggesting that protein prenylation contributes to suppression of the SASP by simvastatin.

### Rac1 and Cdc42 contribute to suppression of the SASP by simvastatin

**Figure 3:**
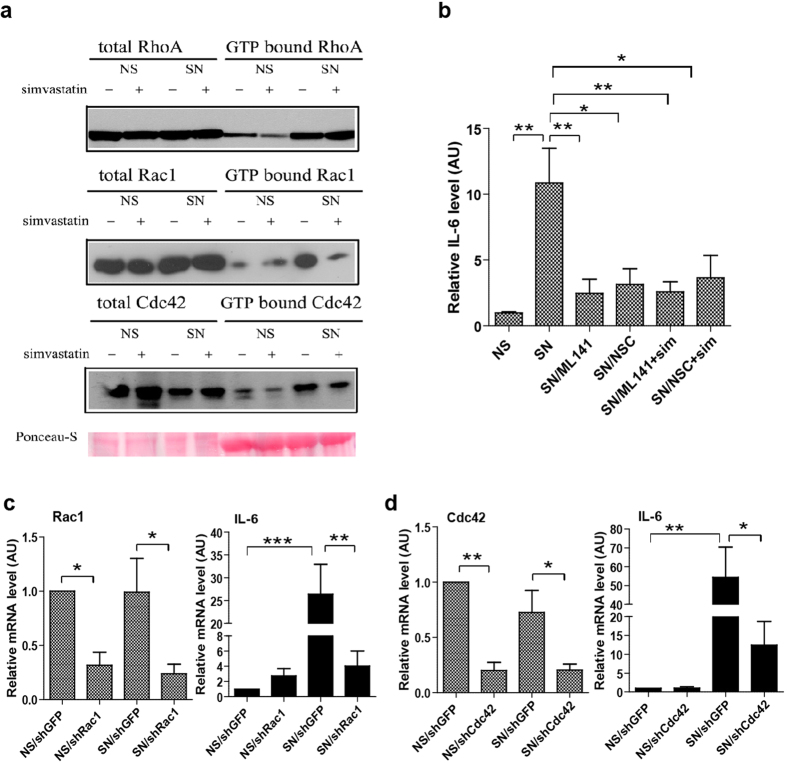
Inhibition of Rac1 or Cdc42 contributes to the suppression of IL-6 expression by simvastatin. (**a**) NS or SN fibroblasts were treated with DMSO or 250 nM simvastatin for 9 d. Whole cell lysates were collected and analyzed for RhoA/Rac1/Cdc42 activation using a commercial kit. GTP bound RhoA/Rac1/Cdc42 are activated forms of the proteins. Ponceau-S staining served as a loading control. The blots were run under the same experimental conditions. The full blots are shown in Supplementary Figure 7. (**b**) HCA2 fibroblasts were irradiated, immediately treated with 5 μM of the Cdc42 inhibitor (ML141), 20 μM of the Rac1 inhibitor (NSC), or co-treated with simvastatin, and allowed to develop a SASP for 9 d. CM were collected and analyzed by ELISA for IL-6 as described above. (**c**) NS or SN fibroblasts were infected with lentiviruses expressing an shRNA against Rac1 (shRac1) or a control shRNA (shGFP), and selected. 9 d later, mRNA was collected for qPCR analysis of Rac1 (left panel) or IL-6 (right panel) mRNA. Actin was used as a control for RNA quantity. (**d**) NS or SN fibroblasts were infected with lentiviruses expressing an shRNAs against Cdc42 (shCdc42) or a control shRNA (shGFP), and selected. 9 d later, mRNA was collected for qPCR analysis for the expression of Cdc42 (left panel) or IL-6 (right panel). Actin was used as a control for RNA quantity. (*p < 0.05, **p < 0.01,***p < 0.001).

Protein prenylation is critical for activation of the Rho family of GTPases. Among these GTPases, Rho A, Rac1 and Cdc42 are the most well studied and have been linked to cellular senescence^26^,^27^. We therefore examined total protein levels of Rho A, Rac1 and Cdc42, as well as levels of their GTP bound active forms, in senescent fibroblasts. The total protein levels of all three Rho GTPases remained similar in simvastatin- and vehicle-treated non-senescent and senescent cells (Fig. 3a). However, there was significantly more GTP bound Rac1 and Cdc42 in vehicle-treated senescent cells compared to simvastatin-treated cells (Fig. 3a).

To test whether activation of Rac1 or Cdc42 is required for IL-6 secretion by senescent cells, we added the Rac1 inhibitor NSC, or Cdc42 inhibitor ML141, to senescent cells and examined IL-6 levels in CM by ELISA. Either inhibitor reduced IL-6 levels by >75% (Fig. 3b), without affecting the growth arrest (Supplementary Figure 4). Furthermore, simvastatin plus either inhibitor did not show an additive effect, suggesting that simvastatin reduces IL-6 secretion by inhibiting Rac1 or Cdc42 activation. To confirm that Rac1 or Cdc42 activation is essential for IL-6 secretion by senescent cells, we assessed IL-6 mRNA levels in senescent cells after depleting Rac1 or Cdc42. shRNAs targeting Rac1 or Cdc42, but not a control shRNA (shGFP), reduced Rac1 or Cdc42 mRNA levels respectively and, in both cases, significantly suppressed IL-6 mRNA levels (Fig. 3c,d). These finding support the idea that Rac1 and Cdc42 activation contribute to suppression of the SASP by simvastatin.

### Simvastatin suppresses the cancer-promoting effects of senescent cells

**Figure 4:**
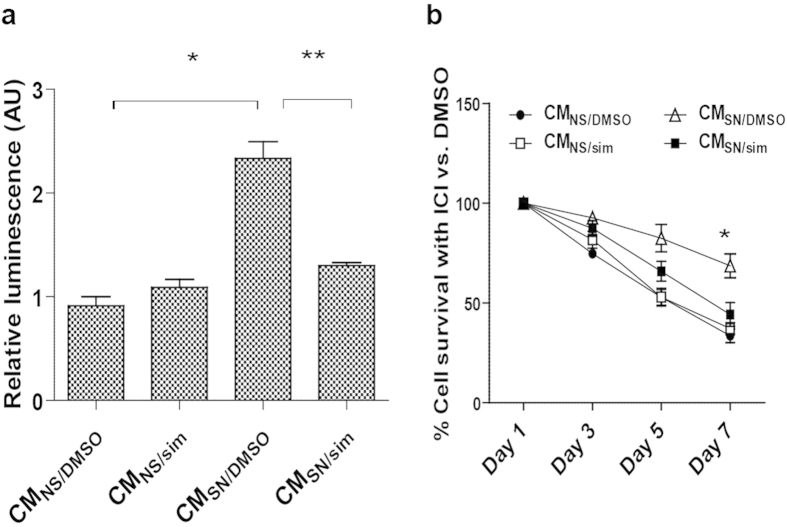
Simvastatin suppresses SASP-induced breast cancer cell proliferation. (**a**) Conditioned media were collected from DMSO or simvastatin treated non-senescent (CM_NS/DMSO_, CM_NS/sim_) or senescent (CM_SN/DMSO_, CM_SN/sim_) HCA2 cells as described above. MCF7 breast cancer cells were kept in the indicated CM containing 0.5% FBS for 48 hrs. Cell viability/proliferation was examined using a commercial kit. (**b**) CM were collected as described in (**a**), and applied to MCF7 cells treated with DMSO or the estrogen receptor antagonist ICI 182780 (ICI) for the indicated times. The percentage of cell survival (ICI vs. DMSO) was determined using crystal violet staining. (*p < 0.05, **p < 0.01).

Senescent cells can stimulate cancer cell proliferation in a paracrine manner through the SASP^5^,^11^. To determine whether simvastatin mitigates this effect, we measured the ability of CM from non-senescent or senescent cells, treated with either simvastatin or vehicle, to stimulate the viability and proliferation of MCF7 human breast cancer cells. CM from senescent cells stimulated by 2-fold the viability/proliferation of MCF7 cells compared to CM from non-senescent cells (Fig. 4a). Importantly, simvastatin suppressed this stimulatory activity, indicating that simvastatin mitigates the cell non-autonomous pro-malignant activities of the SASP. This finding was confirmed using another breast cancer cell line ZR-75 (Supplementary Figure 5a).

The estrogen receptor (ER) antagonist ICI 182780 (Fulvestrant) has been used to treat ER-positive breast cancer in postmenopausal women^39^, and inhibits MCF7 cell proliferation in culture and *in vivo*^40^. To determine whether senescent cells affected the breast cancer cell response to ICI 182780, and whether simvastatin altered the effects, we tested the viability/proliferation of MCF7 cells after adding ICI 182780 to the CM of non-senescent or senescent cells that were treated with simvastatin or vehicle. After 7 d, ICI 182780 killed about 50% of MCF7 cells when the cells were cultured in CM from non-senescent cells treated with simvastatin or vehicle (Fig. 4b). When cultured in CM from vehicle-treated senescent cells, ICI 182780 killed only about 25% of MCF7 cells after 7 days, indicating that senescent CM confers resistance to ICI 182780 killing. Importantly, the survival rate returned to 50% when MCF7 cells were maintained in CM from simvastatin-treated senescent cells during ICI 182780 treatment. Thus, senescent cells might contribute to anti-estrogen resistance in breast cancer patients, and simvastatin can mitigate this activity.

### Simvastatin suppresses SASP-induced activation of the ERK pathway in breast cancer cells

**Figure 5:**
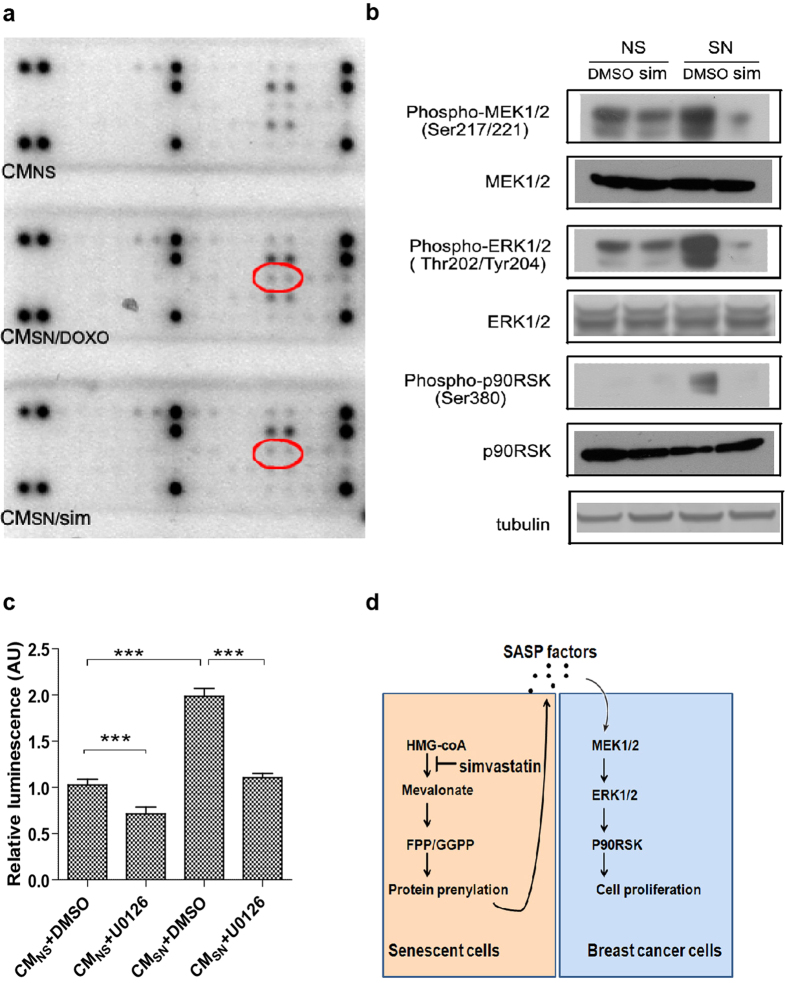
Simvastatin suppresses the SASP-induced activation of the ERK pathway in breast cancer cells. (**a**) MCF7 cells were cultured in CM from NS, DMSO- or simvastatin-treated SN cells for 24 hrs. Changes in the phosphorylation status of proteins belonging to receptor tyrosine kinase pathways were determined using a commercial kit. (**b**) Conditioned media were collected from DMSO- or simvastatin-treated NS or SN cells as described above. MCF7 breast cancer cells were cultured in the indicated CM containing 0.5% FBS for 24 hrs. Whole cell lysates were collected and analyzed by western blotting. Tubulin served as a loading control. The blots were run under the same experimental conditions. The full blots are shown in Supplementary Figure 8. (**c**) Conditioned media were collected from non-senescent (CM_NS_) or senescent (CM_SN_) HCA2 cells as described above. MCF7 breast cancer cells were cultured in the indicated CM containing 0.5% FBS with DMSO or 10 μM U0126 for 48 hrs. Cell viability/proliferation was examined using a commercial kit. (**d**) Model depicting the molecular pathways through which simvastatin suppresses SASP-induced breast cancer cell proliferation. (***p < 0.001).

To determine the signal transduction pathways through which senescent cell CM modulate MCF7 cell proliferation, we cultured MCF7 cells with CM from non-senescent cells or vehicle- or simvastatin-treated senescent cells for 24 hrs, and used receptor tyrosine kinase (RTKs) antibody arrays to assess the activation of a wide range of signaling molecules that are downstream of RTKs, which control cell survival, proliferation, growth and metabolism^41^. Dysregulated RTK signaling has been implicated in a variety of cancers, including breast cancer^42^. From the array, phospho-ERK1/2 (Thr202/Tyr204, red circle) was the only phosphoprotein that was induced in MCF7 cells by CM from vehicle-treated senescent cells, and was reduced in MCF7 cells by CM from simvastatin-treated senescent cells (Fig. 5a).

Activation of the ERK1/2 pathway has been linked to poor responses to hormone therapy in some breast cancer patients^43^. To confirm activation of the ERK1/2 pathway in breast cancer cells by CM from senescent cells, we examined the phosphorylation status of ERK1/2 and several other kinases that are part of the pathway. Consistent with the antibody array analyses, phospho-ERK1/2 (Thr202/Tyr204), as well as phospho-MEK1/2 (Ser217/221) and phospho-p90RSK (Ser380), were activated in both MCF7 and ZR-75 cells by CM from senescent cells (Fig. 5b; Supplementary Figure 5b). Importantly, these activations were strongly reduced in MCF7 and ZR-75 cells treated with CM from simvastatin-treated senescent cells (Fig. 5b; Supplementary Figure 5b).

To test whether activation of the ERK1/2 pathway contributes to the cancer promoting effect of the SASP, we treated MCF7 and ZR-75 cells with the MEK1/2 inhibitor U0126 (or vehicle) together with CM from non-senescent or senescent cells. Senescent cell CM stimulated the viability/proliferation of MCF7 and ZR-75 cells 2-fold compared to CM from non-senescent cells (Fig. 5c; Supplementary Figure 5c), while U0126 suppressed this stimulatory activity. To test whether IL-6, one of the main SASP factors, contributes to the cancer promoting effect of the SASP and the ERK1/2 pathway activation, we added recombinant IL-6 to CMns-treated MCF7 cells and an anti-IL-6 antibody to CMsn-treated MCF7 cells. Recombinant IL-6 slightly increased the viability/proliferation of MCF7 cells and the level of phospho-ERK1/2, phospho-MEK1/2 and phospho-p90RSK (Supplementary Figure 6a and b), while anti-IL-6 antibody slightly suppressed the stimulatory activity of CMsn on the viability/proliferation of MCF7cells and the level of phospho-ERK1/2, phospho-MEK1/2 and phospho-p90RSK (Supplementary Figure 6a and b).

These findings suggest that senescent cells promote breast cancer cell proliferation by activating the ERK1/2 pathway partially through paracrine signaling of IL-6, and that simvastatin mitigates this activity.

## Discussion

Cellular senescence is a well-established tumor-suppressive mechanism that cell autonomously arrests the growth of cells at risk for transformation^1^,^8^,^44^. However, it is also recognized that senescent cells can promote cancer progression in a cell non-autonomous manner through the secretion of growth factors, cytokines, and chemokines (SASP)^11^,^45^,^46^. Cellular senescence thus acts as a double-edged sword during tumorigenesis.

Radiation therapy and chemotherapy induce cellular senescence in normal and cancer cells, and these senescence-inducing therapies are routinely given to patients without considering the cancer-fueling risk of cellular senescence^47^. To achieve optimal anti-cancer benefits of these therapies, it might be important to develop adjuvant therapies that selectively suppress the cancer-promoting SASP without affecting the cancer-suppressing growth arrest. Here, we show that simvastatin, a widely prescribed cholesterol-lowering drug, is a potential candidate for such a therapy. We found that simvastatin reduces the expression of IL-6 and other SASP factors without affecting many other senescence-associated phenotypes, including the growth arrest. As a consequence, CM from simvastatin-treated senescent fibroblasts did not stimulate breast cancer cell proliferation compared to CM from vehicle-treated senescent fibroblasts.

It is also noteworthy that senescent CM promoted resistance to Fulvestrant (ICI 182780) in breast cancer cells, while simvastatin-treated senescent CM had no such effect. Hormone therapy, which aims to block estrogen receptor (ER) action, is the most effective targeted treatment for ER-positive breast cancer, but the prevalence of intrinsic and acquired resistance often limits its success^48^. Several mechanisms have been proposed to account for this resistance, including deregulation of the ER pathway, alterations in apoptosis and cell cycle regulation, and hyper-activation of multiple pro-proliferation pathways^33^,^49^. In addition, there is increasing evidence that the tumor microenvironment is a critical determinant of breast cancer progression and response to therapies, including endocrine therapy^50^. Senescent fibroblasts in the microenvironment have been shown to promote tumorigenesis and cancer cell proliferation^11^,^45^,^46^,^51^. Our study now suggests a potential contribution of senescent fibroblasts to endocrine resistance in breast cancer, and we further demonstrate that this contribution can be suppressed by reducing the SASP.

We also found that the pro-proliferative effect by the SASP might act through activation of the MEK-ERK1/2-RSK pathway in breast cancer cells. Whereas several inhibitors targeting kinases that belong to this pathway have been approved by the FDA for the treatment of melanoma, hyperactivation of this pathway has also been linked to endocrine resistance in breast cancer^52^,^53^. Therefore, it might be possible to mitigate the SASP-induced endocrine resistance with inhibitors of the MEK-ERK1/2 pathway. Adding anti-IL-6 antibody to CMsn-treated MCF7 cells slightly reduced the activation of the MEK-ERK1/2-RSK pathway, suggesting that IL-6 might be one of the SASP factors that activate this pathway.

The SASP affects cancer cell phenotypes in complex ways. It not only promotes cancer progression, but also reinforces the cancer-suppressing growth arrest in a paracrine manner^4^,^6^, and attracts innate immune cell to activate immune surveillance^54^. Therefore, in order to develop effective pro- or anti-SASP therapies, it will be important to understand the complete SASP profile, its tissue- and spatiotemporal specificity and the signaling pathways that regulate it.

We showed that simvastatin reduces the SASP through inhibition of protein prenylation and consequent suppression of Rac1 and Cdc42 activities. Rac1 and Cdc42 are Rho GTPase family members that regulate actin dynamics and cell proliferation^55^. There are conflicting reports on the role of Rac1 in cellular senescence. One report showed that repression of Rac1 activity by CDK5 was required for senescent phenotypes^56^, and depletion of Rac1 inhibited CCN1-induced senescence, possibly by reducing ROS^57^. However, another report showed that constitutive Rac1 activation induced mitochondrial oxidative stress and premature senescence^58^. Yet another report showed that either gain or loss of Rac1 activity resulted in cellular senescence^26^. It is therefore possible that Rac1 activity must be maintained within a narrow range, and abnormal Rac1 activity, either gain or loss of function, can result in cellular senescence.

Increased Cdc42 activity may be involved in normal aging. Active Cdc42 levels are significantly higher in several tissues of aged mice compared to younger mice^59^, and elevated Cdc42 activity has been causally linked to the aging of hematopoietic stem cells^60^. Cdc42 activation was reported to be sufficient to promote p53-dependent premature cellular senescence^59^, and senescence-associated inflammation^27^. We found that Rac1 or Cdc42 depletion suppressed IL-6 secretion by senescent fibroblasts, without reversing the senescence growth arrest.

In summary, we show that simvastatin suppresses the SASP and its cancer-promoting effects by repressing Rac1 and Cdc42 activation. We suggest that the SASP stimulates breast cancer cell proliferation through activation of the MEK-ERK1/2 pathway, and that simvastatin suppresses this SASP-induced activation in breast cancer cells (as schematically shown in Fig. 5d). Our data also suggest that the SASP might confer endocrine resistance to breast cancer cells, which can be abrogated by simvastatin.

## Methods

### Cell culture

HCA2 are normal human neonatal foreskin fibroblasts (originally obtained from O. Pereira-Smith, University of Texas Health Science Center, San Antonio, TX), WI-38 are normal human embryonic lung fibroblasts (obtained from the American Type Culture Collection), and MCF7 and ZR-75 are human breast cancer cells (obtained from the American Type Culture Collection). Fibroblasts were cultured in DMEM plus 10% FBS and penicillin/streptomycin at 3% oxygen and 10% CO_2_. Breast cancer cells were cultured in DMEM plus 10% FBS and penicillin/streptomycin at 20% oxygen and 10% CO_2_.

### Senescence induction, simvastatin treatment, SA-β-Gal and BrdU labeling

Senescence was induced by exposing subconfluent cultures to 10 Gy X-rays (IR); control cultures were mock irradiated. Non-senescent (NS) cells were used 1–2 d after mock irradiation or infection; senescent (SN) cells were used 7–10 d after irradiation or infection. Cells were considered NS if >70% incorporated BrdU over 2 days and <10% expressed SA-β-Gal, and SN if <10% incorporated BrdU and >80% expressed SA-β-Gal. Simvastatin was added to the cells immediately after IR, and maintained the cells in medium containing the drug for 9 d. Medium was changed every other day. On the 9th day, cells were incubated in serum-free medium without simvastatin and the conditioned medium (CM) was collected 24 hrs later. We detected SA-β-Gal as described^2^ using a commercial kit (BioVision), and DNA synthesis using a modified BrdU labeling kit (Roche) or EdU proliferation assay (Life Technologies).

### Real-time quantitative PCR

Cells were lysed and reverse transcribed using iScript™ Reverse Transcription Supermix for RT-qPCR (Bio-Rad). Quantitative PCR (qPCR) was performed using SensiFAST™ SYBR® No-ROX Kit (Bioline) and following manufacturer’s instructions. Primer sequences for qPCR are listed in Supplementary Table 1.

### Western blotting

Cells were lysed in RIPA buffer, lysates were sonicated (10 s) and clarified by centrifugation, and protein concentrations were measured using Bradford reagents. Samples were incubated at 95 °C for 10 min, loaded on 4–15% gradient tris-glycine SDS-polyacrylamide gels (Invitrogen), separated by electrophoresis and transferred to PVDF membranes. Membranes were blocked in TBST/BSA for 1 h at room temperature, probed overnight at 4 °C with primary antibodies in blocking buffer, washed in TBST and incubated with horseradish peroxidase-conjugated secondary antibodies for 1 h at room temperature. Signals were detected using Supersignal® West chemiluminescent substrate (Thermo Scientific).

### Viral vectors and infection

Lentiviruses encoding shRNAs against GFP (control) and HMG-CoA reductase, FNTA, Rac1 and Cdc42 were from Open Biosystems. The lentiviral NF-kB reporter-luciferase construct was from SA Biosciences. Lentiviruses were produced and used as described^5^. To limit side effects of infection, viral titers were adjusted to infect 80–90% of cells, and cultures were subsequently selected in puromycin for 3 d.

### ELISAs

Conditioned media were collected, filtered and stored at –80°C, and cells remaining on the culture dish were counted for normalization. ELISAs were performed using a kit and procedures from PerkinElmer (IL-6 AL223F).

### Rho GTPase activity assays

RhoA, Rac1 and Cdc42 activities were determined using rhotekin-RBD, which specifically binds activated Rho, and PBD-PAK, which has a high affinity for both GTP-Rac and GTP-Cdc42, using the RhoA/Rac1/Cdc42 Activation Assay Combo Biochem kit (Cytoskeleton). Briefly, cells were lysed with cold buffer containing 50 mM Tris-HCl pH 7.4, 2 mMMgCl_2_, 1% Nonidet P-40, 10% glycerol, 100 mM NaCl, and protease inhibitor mixture (Roche Diagnostics). Lysates were clarified by centrifugation for 5 min at 14,000 × *g*, and 500 μg of total cell protein were processed using 50 μg of rhotekin-RBD beads and 20 μg of PAK-PBD beads, rotating for 60 min at 4 °C. The beads were washed with lysis buffer and heated for 5 min at 100 °C in SDS-PAGE sample buffer, then analyzed for bound RhoA, Rac1, and Cdc42 molecules by western blotting using anti-RhoA (1:500, Cytoskeleton), anti-Rac1 (1:500, Cytoskeleton) or anti-Cdc42 (1:500, Cytoskeleton) antibodies.

### Antibody Arrays

Cells were lysed and lysates processed as described for western blotting. Protein expression and phosphorylation status of receptor tyrosine kinases pathway components were determined by the PathScan® RTK Signaling Antibody Array Kit (Cell signaling), following the manufacturer’s instructions.

### Cell viability assays

Cells were plated in 96-well plates and 2 d later were serum-starved for 24 h. Cells were then incubated with conditioned media and/or drugs for an additional 48 h before cell viability/proliferation assays were performed using the CellTiter-Glo® Luminescent kit (Promega) according to the manufacturer’s instructions.

### Statistical Analysis

Error bars on all graphs represent the standard error of the mean from at least three independent experiments. The p values on multiple comparisons were calculated using one-way ANOVA with Bonferroni posttest.

## Additional Information

**How to cite this article**: Liu, S. *et al.* Simvastatin suppresses breast cancer cell proliferation induced by senescent cells. *Sci. Rep.* **5**, 17895; doi: 10.1038/srep17895 (2015).

## Supplementary Material

Supplementary Information
