# Supplementary material for: Simvastatin suppresses breast cancer cell proliferation induced by senescent cells
Source: Sci Rep. 2015 Dec 14;5:17895. doi: 10.1038/srep17895 (PMC4677323; doi:10.1038/srep17895)
Supplement: Supplementary Information [file srep17895-s1.doc]

**Simvastatin suppresses breast cancer cell proliferation induced by senescent cells**

Su Liu^1^, Harpreet Uppal^2^, Marco Demaria^1^, Pierre-Yves Desprez^1,3^, Judith Campisi^1,4,*^, and Pankaj Kapahi^1,*^

^1^Buck Institute for Research on Aging, Novato, CA 94945, USA

^2^Santa Rosa Junior College, Santa Rosa, CA 95401, USA

^3^California Pacific Medical Center, Research Institute, San Francisco, CA 94107, USA

^4^Life Sciences Division, Lawrence Berkeley National Laboratory, Berkeley, CA 94720, USA

^*^Correspondence:

Pankaj Kapahi, Buck Institute for Research on Aging, 8001 Redwood Boulevard, Novato, CA 94945, USA; Telephone: 1-415-209-2201; Fax: 1-493-3640; Email: pkapahi@buckinstitute.org; or Judith Campisi, Buck Institute for Research on Aging, 8001 Redwood Boulevard, Novato, CA 94945, USA; Telephone: 1-415-209-2066; Fax: 1-415-493-3640; E-mail: JCampisi@lbl.gov

Running title: Simvastatin reduces the SASP

**Supplementary Figure Legends**

**Supplementary Figure 1. (a)** Diagram describing the procedure collecting conditioned media (CM). **(b)** Senescent (SN) HCA2 fibroblasts were incubated in media containing the indicated concentrations of simvastatin (sim) or vehicle (DMSO). 9 d later, cells that remained on the plate were counted. **(c)** Non-senescent (NS) or senescent (SN) HCA2 fibroblasts were treated with simvastatin (sim) or DMSO at indicated time points after irradiation, and CM were collected and analyzed 9 d later by ELISA for IL-6. Cells that remained on the plate at the time of collection were counted for normalization. **(d)** NS or SN HCA2 fibroblasts were treated with 250 nM simvastatin or DMSO. 9 d later cells were stained for SA-β-gal. **(e)** NS or SN HCA2 fibroblasts were treated with 250 nM simvastatin or DMSO. 8 d later, cells were given BrdU for 24 hrs, fixed and immunostained for nuclear BrdU. **(f)** NS or SN cells described in **(d)** were fixed and stained for 53BP1 foci.

**Supplementary Figure 2. (a)** NS or SN WI-38 fibroblasts were incubated in media containing 250 nM simvastatin (sim) or DMSO. The cells were given simvastatin or DMSO immediately after irradiation and analyzed 9 d later. Conditioned media were analyzed by ELISA for IL-6 secretion as described in Figure 1. **(b)** NS or SN WI-38 fibroblasts were given 250 nM simvastatin or DMSO. 9 d later, RNA was extracted for quantitative PCR analysis of the indicated mRNAs. Actin was used as a control for RNA quantity. **(c)** NS or SN WI-38 cells described in **(b)** were given BrdU for 24 hrs, fixed and immunostained for nuclear BrdU, and analyzed for the percentage of BrdU-positive cells manually. **(d)** NS or SN WI-38 cells described in **(b)** were stained for SA-β-gal. The percentage of cells that express SA-β-gal was scored manually. (*p<0.05, **p<0.01,***p<0.001)

**Supplementary Figure 3. (a)** HCA2 fibroblasts were infected with vector control (ctrl) or H-Ras. After a 2-d treatment with puromycin, cells were incubated in media containing simvastatin (sim) or vehicle (DMSO). 7 d later, cells were stained for SA-β-gal. **(b)** HCA2 fibroblasts were treated as described in (a). 7 d later, RNA was extracted for quantitative PCR analysis of the indicated genes, which were normalized to actin mRNA.(*p<0.05, **p<0.01,***p<0.001)

**Supplementary Figure 4.** HCA2 fibroblasts were irradiated, immediately treated with 5 μM of the Cdc42 inhibitor (ML141) or 20 μM of the Rac1 inhibitor (NSC). Cell number was counted 9 d after irradiation. The original seeding number was 250,000.

**Supplementary Figure 5. (a)** Conditioned media were collected from DMSO- or simvastatin-treated non-senescent (CM_NS/DMSO_, CM_NS/sim_) or senescent (CM_SN/DMSO_, CM_SN/sim_) HCA2 cells as described above. ZR-75 breast cancer cells were cultured in the indicated CM containing 0.5% FBS for 48 hrs. Cell viability/proliferation was examined using a commercial kit. **(b)** Conditioned media were collected from DMSO- or simvastatin-treated NS or SN HCA2 cells as described above. ZR-75 breast cancer cells were cultured in the indicated CM containing 0.5% FBS for 24 hrs. Whole cell lysates were then collected and analyzed by Western blotting. Tubulin served as a loading control. **(c)** Conditioned media were collected from non-senescent (CM_NS_) or senescent (CM_SN_) HCA2 cells as described above. ZR-75 breast cancer cells were cultured in the indicated CM containing 0.5% FBS and DMSO or 10 μM U0126 for 48 hrs. Cell viability/proliferation was examined using a commercial kit. (***p<0.001)

**Supplementary Figure 6. (a)** Conditioned media were collected from non-senescent (CM_NS_) or senescent (CM_SN_) HCA2 cells as described above. MCF7 breast cancer cells were cultured in the indicated CM containing 0.5% FBS with 0.04 ng/ml recombinant IL-6 or 2 µg/ml IL-6 neutralizing antibody for 48 hrs. Cell viability/proliferation was examined using a commercial kit. **(b)** Conditioned media were collected from NS or SN cells as described above. MCF7 breast cancer cells were cultured in the indicated CM containing 0.5% FBS for 2 hrs. Whole cell lysates were collected and analyzed by Western blotting. Tubulin served as a loading control. (*p<0.05, ***p<0.001)

**Supplementary Figure 7.** Supplementary full scans of western blots of the total and GTP bound Rho A, Rac 1 and Cdc42 in Figure 3a. Ponceau-S staining served as a loading control. Full scans were cropped and used in the indicated primary figures.

**Supplementary Figure 8.** Supplementary full scans of western blots of phospho- MEK1/2, MEK1/2, phospho-ERK1/2, ERK1/2, phospho-p90RSK, p90RSK, and tubulin in Figure 5b. Full scans were cropped and used in the indicated primary figures.

**Supplementary Figure 1.**

**
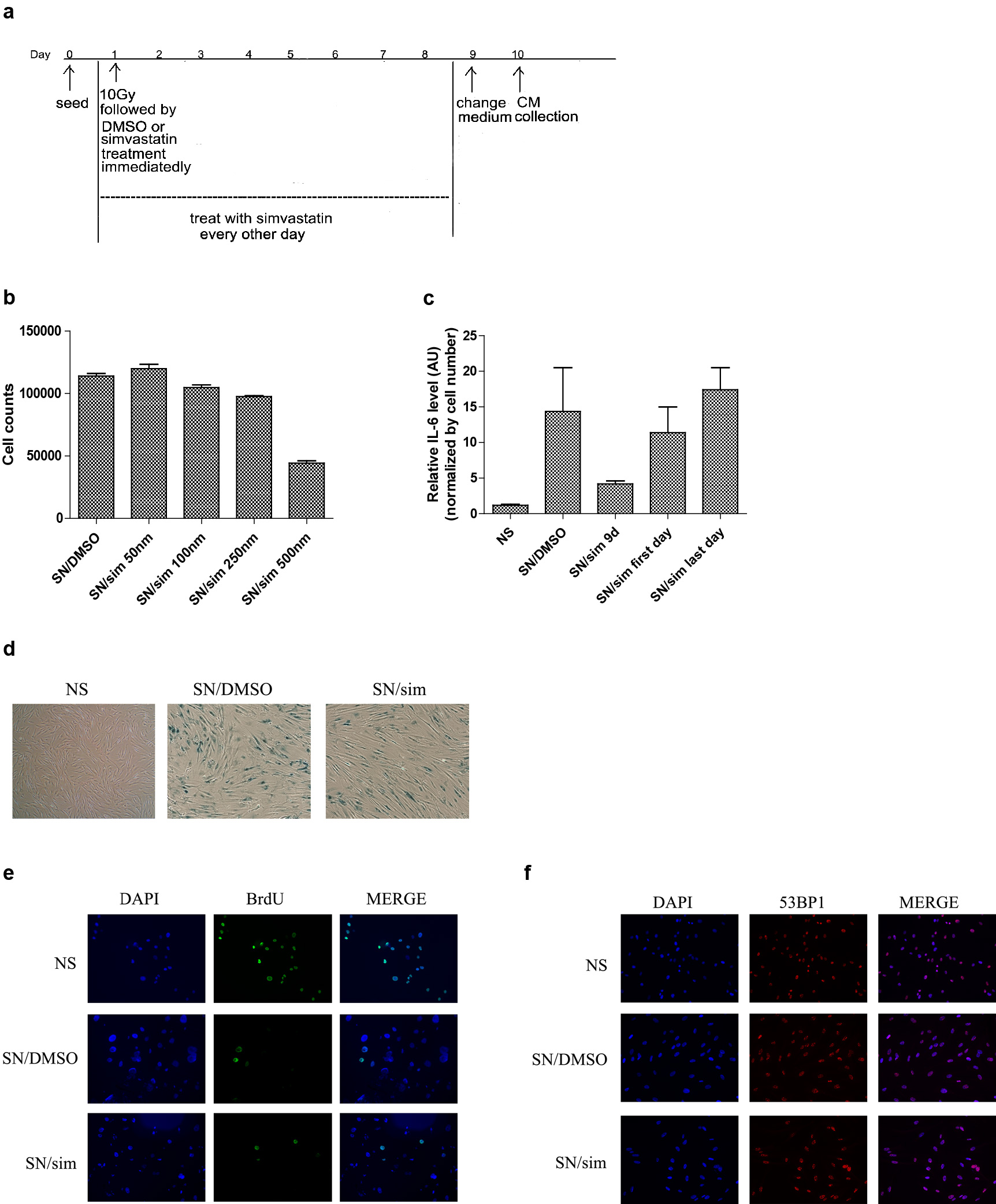
**

**Supplementary Figure 2.**

**
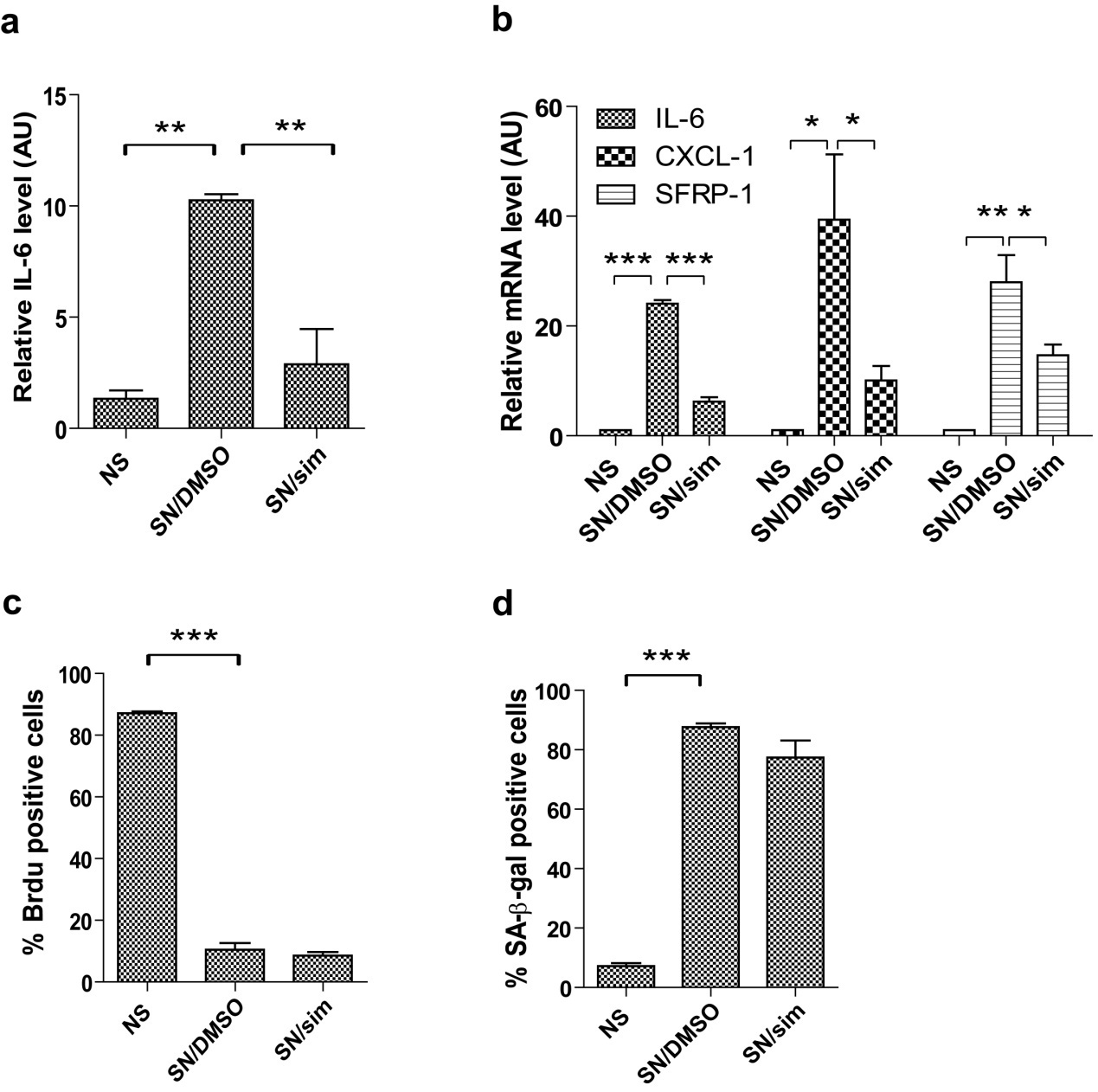
**

**Supplementary Figure 3.**

**
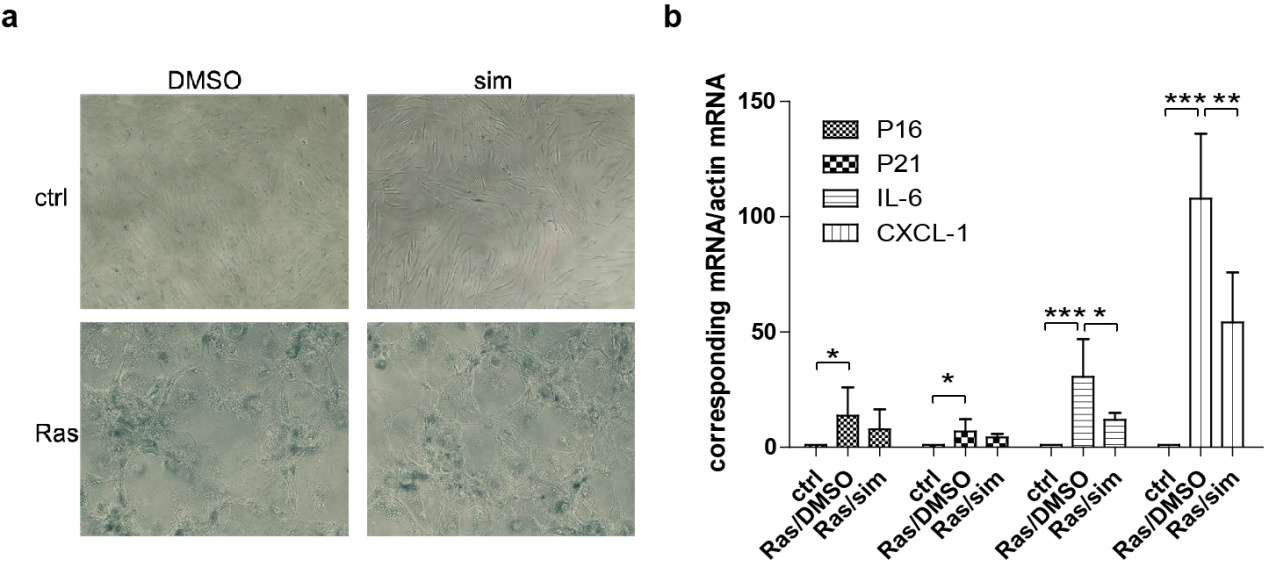
**

**Supplementary Figure 4.**

**
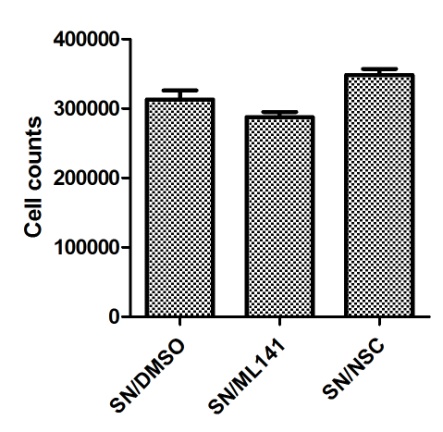
**

**Supplementary Figure 5.**


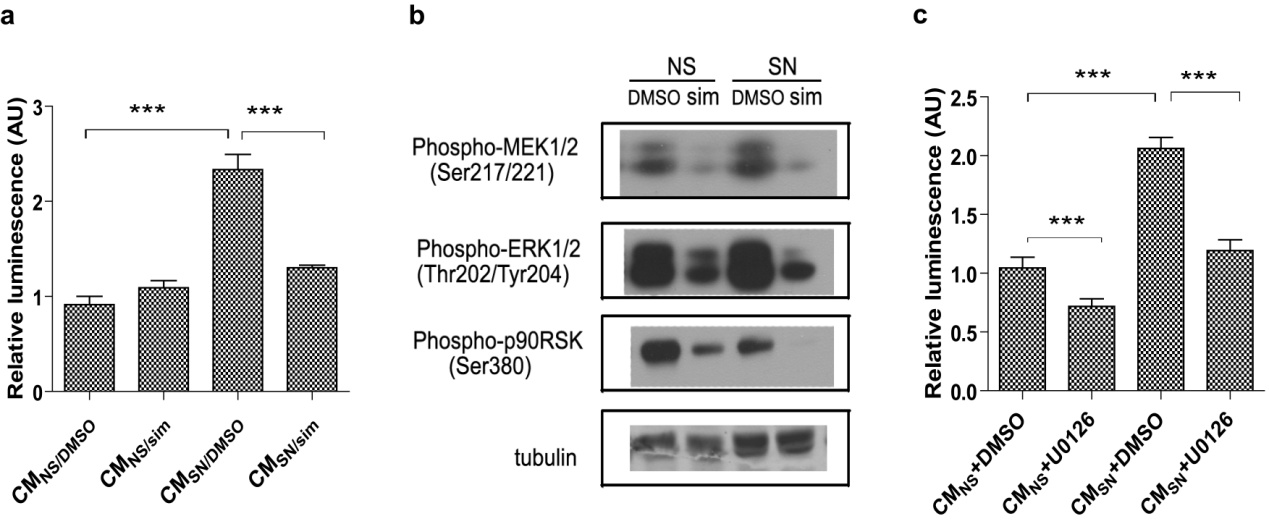


**Supplementary Figure 6.**


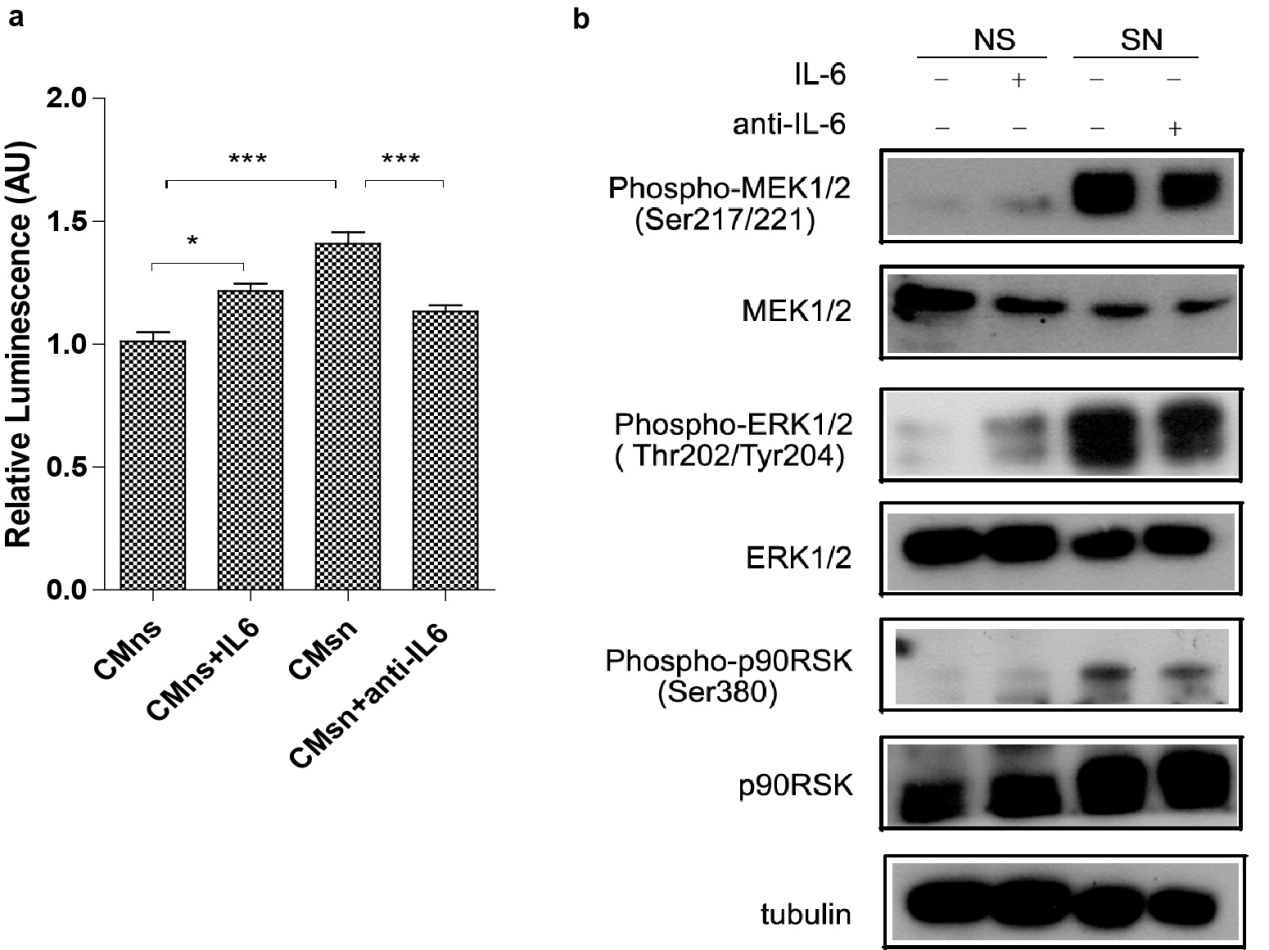


**Supplementary Figure 7.**


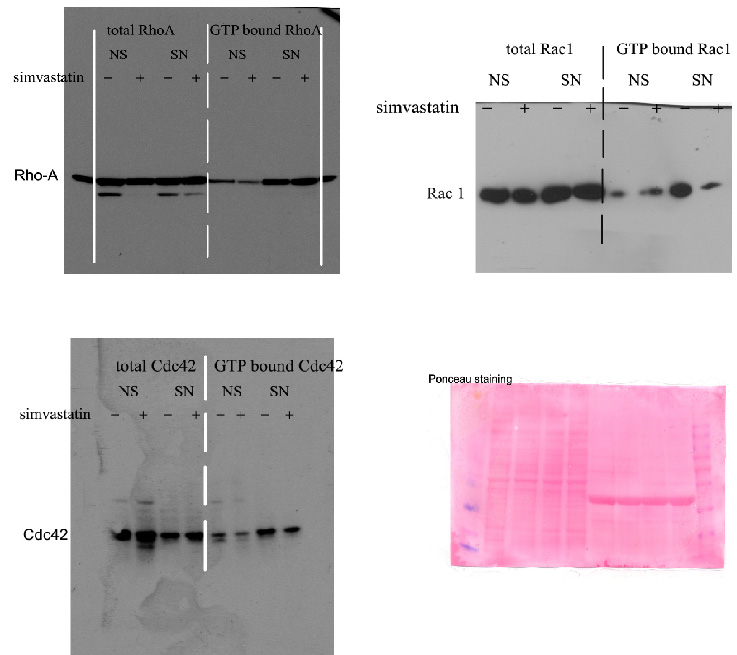


**Supplementary Figure 8.**

**
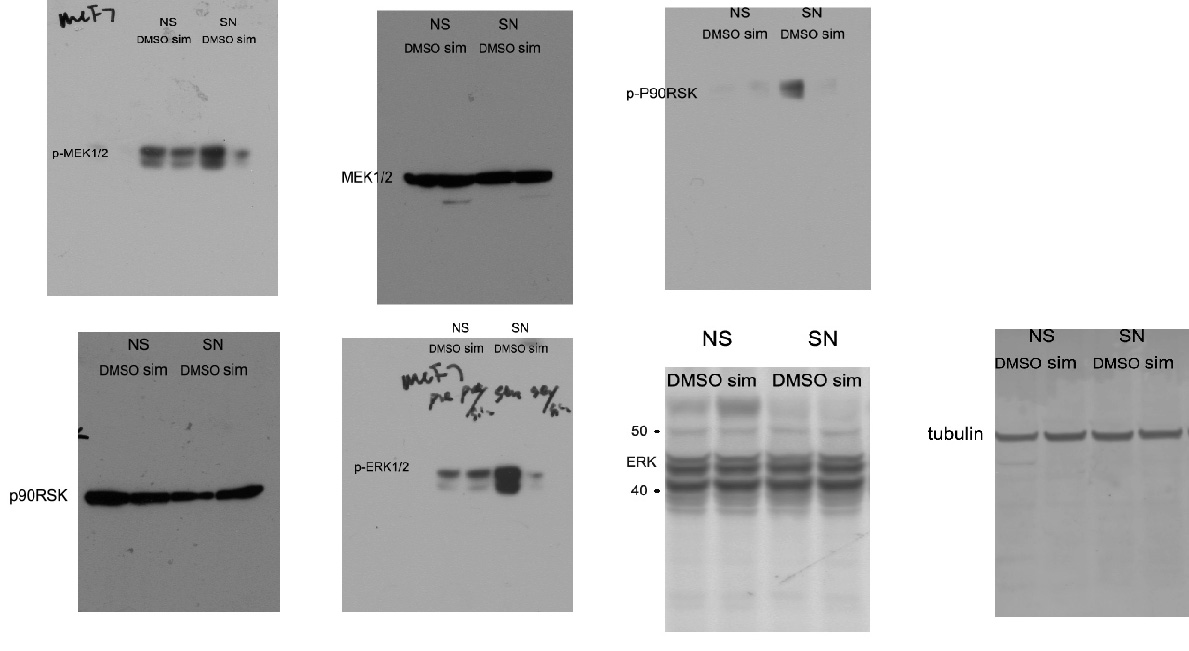
**

**Supplementary Table 1: Sequences for RT-PCR primers used in this study.**

| **Primer name** | **Sequence** |
| --- | --- |
| IL-6 Forward Primer | AACCTGAACCTTCCAAAGATGG |
| IL-6 Reverse Primer | TCTGGCTTGTTCCTCACTACT |
| IL-1α Forward Primer | GAATGACGCCCTCAATCAAAGT |
| IL-1α Reverse Primer | TCATCTTGGGCAGTCACATACA |
| IL-3Forward Primer | CAGACAACGCCCTTGAAGACA |
| IL-3Reverse Primer | GCCCTGTTGAATGCCTCCA |
| IL-8Forward Primer | ACTGAGAGTGATTGAGAGTGGAC |
| IL-8Reverse Primer | AACCCTCTGCACCCAGTTTTC |
| CXCL1 Forward Primer | AGGGAATTCACCCCAAGAAC |
| CXCL1 Reverse Primer | ACTATGGGGGATGCAGGATT |
| RANTES Forward Primer | CCA GCA GTC GTC TTT GTC AC |
| RANTES Reverse Primer | CTC TGG GTT GGC ACA CAC TT |
| β-actin Forward Primer | AATCTGGCACCACACCTTCTAC |
| β-actin Reverse Primer | ATAGCACAGCCTGGATAGCAAC |
| HMGCR Forward Primer | TGATTGACCTTTCCAGAGCAAG |
| HMGCR Reverse Primer | CTAAAATTGCCATTCCACGAGC |
| FNTA Forward Primer | ATCCCGTGGTCCAGATCATTT |
| FNTA Reverse Primer | AAGCTCGTTCACTTCTTTCATCA |
| Cdc42 Forward Primer | GCTGGTGTCGGCATCATACT |
| Cdc42 Reverse Primer | TATGGGCCTTGTCTCACACG |
| Rac1 Forward Primer | AAACCGGTGAATCTGGGCTT |
| Rac1 Reverse Primer | AGAACACATCTGTTTGCGGA |
